# Differential formative effects of service-learning in early psychology training: ethical, socioemotional and technical competences in a Chilean context

**DOI:** 10.3389/fpsyg.2026.1916584

**Published:** 2026-09-11

**Authors:** Ana M. Ruiz-Ortega, Julia Cubillos Romo

**Affiliations:** Department of Social Sciences and Humanities, Psychology Program, Universidad de Aysén, Coyhaique, Chile

**Keywords:** early professional training, formative competences, Latin America, mixed-methods design, professional ethics, psychology education, service-learning, socioemotional competences

## Abstract

Service-learning is widely recognized as an active pedagogy in higher education, yet evidence on its differential effects across distinct formative dimensions, particularly in the early stages of professional training, remains limited. This study examined second-year psychology students’ perceptions of service-learning experiences implemented in schools and social organizations at a regional university in southern Chile. Using a convergent mixed-methods design, a 57-item Likert-type survey covering 10 formative dimensions was combined with four open-ended questions analyzed through inductive thematic analysis (*N* = 55). Quantitatively, ethical and professional responsibility and context and feasibility obtained the highest mean scores (both *M* = 4.50 on a 1–5 scale), followed by critical reflection (*M* = 4.25), whereas conceptual learning (*M* = 4.16) and psychosocial intervention (*M* = 3.79) received comparatively more moderate ratings. Thematic analysis identified five interconnected themes: theory-practice articulation, teamwork and socioemotional skills, ethical sensitization and empathy, contextual understanding of psychosocial problems, and initial approximation to the professional role, alongside recurring expressions of technical insecurity and recognition of training limits. The integration of both data sources suggests that service-learning is a particularly powerful device for fostering ethical dispositions, socioemotional competences, critical reflection and community engagement in early training, while its contribution to disciplinary knowledge and technical intervention competences is more moderate and dependent on specific pedagogical conditions. These findings have direct implications for the curricular design of service-learning in psychology programs, especially regarding the balance between ethical-relational outcomes and the consolidation of more advanced technical competences. Given the single-group post-intervention design and reliance on self-report measures, these findings reflect students’ perceived learning and self-evaluations, highlighting key curricular considerations for service-learning in psychology education.

## 1 Introduction

Active learning methodologies have gained prominence in higher education because of their potential to move beyond the passive transmission of content and to promote more complex and integrated forms of learning that articulate cognitive, attitudinal and relational dimensions (Bonwell and Eison, 1991; Prince, 2004). Within this framework, service-learning has become a strategy that intentionally integrates academic objectives, community participation and critical reflection, creating a situated and relational learning environment that is particularly relevant for the initial training of professionals (Bringle and Hatcher, 1996; Furco, 1996). Its relevance lies in the possibility of activating forms of learning that mobilize ethical, socioemotional and reflective dispositions in real-world contexts, which are difficult to reproduce in conventional classrooms.

International frameworks have emphasized the need for undergraduate programs in health and social science disciplines to integrate psychological knowledge, scientific thinking, interpersonal skills, ethical reflection and progressive professional development throughout the curriculum (American Psychological Association, 2023; Rodolfa et al., 2005). In the Chilean context, proposals have also been advanced to orient psychology education from a competency-based perspective that articulates disciplinary knowledge, professional skills and ethical commitment to the surrounding context (Cabrera et al., 2010). From this perspective, psychological training cannot be reduced to the transmission of theoretical or technical content; it requires situated experiences that foster a contextual understanding of psychosocial problems and the progressive development of the professional role.

Meta-analyses in higher education have documented positive effects of service-learning on academic learning, personal development, civic engagement and interpersonal skills (Celio et al., 2011; Conway et al., 2009; Yorio and Ye, 2012). However, recent reviews have noted that these effects are not homogeneous: service-learning tends to show more robust results in attitudinal and socioemotional dimensions than in cognitive indicators or technical competences (Camilli Trujillo et al., 2021; Narong and Hallinger, 2023; Tijsma et al., 2020). This distinction is crucial for the pedagogical design of service-learning as an active methodology in professional training contexts, where objectives of different nature coexist: on the one hand, the development of ethical and relational dispositions, and on the other, the acquisition of disciplinary and technical competences.

Nevertheless, important gaps remain regarding the specific formative mechanisms of service-learning. Although benefits have been documented in general indicators such as satisfaction, civic engagement or social responsibility, there is less evidence on how students interpret socioemotional and ethical learning derived from their participation in community experiences, especially in the early stages of training (Duckworth and Yeager, 2015; Soto et al., 2021). This gap is particularly relevant in disciplines such as psychology, where these competences constitute central domains of professional performance and involve complex constructs that are difficult to capture through standardized self-report instruments. Moreover, if instruments for evaluating service-learning do not distinguish between formative dimensions of different nature, it becomes difficult to identify which aspects of pedagogical design need to be strengthened.

Against this backdrop, the present study addresses four interrelated gaps in the service-learning literature. First, it examines the differential formative effects of service-learning across clearly distinguished dimensions, including conceptual, socioemotional, ethical and technical competences, rather than relying on global satisfaction or undifferentiated outcome scores. Second, it focuses on early stages of psychology training, a disciplinary and developmental moment in which socioemotional and ethical learning may be particularly salient yet remains understudied. Third, it employs a convergent mixed-methods design that systematically integrates quantitative indicators of perceived learning with thematic analysis of students’ narrative accounts, in order to connect patterns of ratings with lived meanings. Fourth, it provides empirical evidence from an underrepresented Latin American context, where service-learning has expanded rapidly but remains less visible in the international research landscape. Accordingly, this study aimed to examine the differential formative contributions of a service-learning course in early psychology training at a regional university in southern Chile. These aims can be summarized in the following research questions:

*RQ1*: What is the pattern of students’ perceptions across the 10 formative dimensions of the service-learning experience in early psychology training?

*RQ2*: What meanings do students attribute to their participation in community-based service-learning interventions?

*RQ3*: How do quantitative and qualitative findings integrate to characterize the scope and limits of service-learning as a formative device in early psychology training?

## 2 Theoretical framework

### 2.1 Service-learning as an active methodology in higher education

Experiential and situated learning methodologies have gained increasing relevance in higher education because of their potential to connect academic training with real-world problems and social interaction settings (Kolb, 1984; Choi et al., 2023). Within this framework, service-learning has been described as a pedagogical methodology that intentionally integrates academic learning, community participation and critical reflection in higher education contexts (Furco, 1996; Jacoby, 2014). Its relevance lies in the possibility of articulating curricular objectives with experiences developed in real social settings, fostering contextualized and relational forms of learning that go beyond the boundaries of the conventional classroom. In the context of professional education, this makes service-learning particularly suitable for articulating early contacts with communities and the gradual assumption of a professional role.

Meta-analyses in higher education have documented positive effects of service-learning on dimensions such as academic learning, personal development, civic engagement and interpersonal skills (Celio et al., 2011; Conway et al., 2009; Yorio and Ye, 2012). These studies show that the benefits range from improvements in attitudes toward society and civic participation to moderate effects on disciplinary knowledge, cognitive development and problem-solving skills. Taken together, this evidence suggests that service-learning can be an effective strategy for promoting complex forms of learning that integrate cognitive, attitudinal and relational components.

However, recent reviews have highlighted substantial conceptual and methodological heterogeneity in the field, which makes it difficult to draw robust conclusions about the formative mechanisms of service-learning and limits comparability across studies (Camilli Trujillo et al., 2021; Narong and Hallinger, 2023; Tijsma et al., 2020). In particular, it has been documented that service-learning tends to generate more robust and consistent effects in attitudinal and civic engagement dimensions than in strictly cognitive indicators or technical competences. This distinction is pedagogically relevant: if the impact of service-learning varies across learning dimensions, it becomes important to identify which dimensions it strengthens more naturally and which ones need to be complemented with other active strategies.

In psychology education, service-learning has been considered particularly pertinent because it offers an intermediate space between academic experience and initial exposure to professional practice (Bringle et al., 2016). These experiences allow students to interact with people, institutions and communities, developing initial understandings of psychological practice and of their emerging professional identity. Recent studies have shown that service-learning can contribute to the development of social responsibility, community engagement, self-efficacy and an initial understanding of the professional role among psychology students, particularly in school and community settings (Sandoval et al., 2025; Valdez and Lovell, 2022). Yet less is known about how these early experiences shape socioemotional and ethical learning in undergraduate psychology, especially in Latin American regional universities.

Nevertheless, mere exposure to community contexts does not guarantee meaningful learning. The formative contributions of service-learning depend on specific pedagogical conditions, including clarity of objectives, curricular integration, strong articulation between theory and practice, systematic teaching supervision and structured spaces for reflection (Eyler and Giles, 1999; Sturgill and Motley, 2014). From critical approaches to service-learning, it has been emphasized that the most meaningful experiences are those that recognize communities as active partners, promote long-term collaborative relationships and avoid assistentialist or extractive approaches, placing ethical and political reflection at the center of the formative process (Gerstenblatt, 2014; Mitchell, 2008). For students in early stages of training, service-learning should therefore be understood as an experience of progressive approximation to professional practice rather than as evidence of fully consolidated competences.

### 2.2 Socioemotional and ethical competences in university training

Psychology education has evolved toward competence-based approaches that emphasize the development of integrated professional competences, articulating knowledge, skills, attitudes, values and reflective capacities needed for contextually grounded professional practice (Fouad et al., 2009; Kaslow et al., 2009). These competence models highlight domains such as ethics, professionalism, reflective practice, interpersonal relationships and sensitivity to diversity as transversal dimensions of professional development across the training continuum. Recent studies with graduates from psychology programs in Europe have shown that students perceive the development of a broad set of disciplinary and generic competences, reinforcing the centrality of competence-based approaches in undergraduate education (Papageorgi et al., 2024). These frameworks provide a useful lens for examining which aspects of professional development may be most strongly activated by service-learning experiences in the early stages of training.

Socioemotional competences have been conceptualized as a set of skills and dispositions related to empathy, emotional regulation, interpersonal communication, perspective taking and effective collaboration (Soto et al., 2021). In psychology, these competences are critical because of the interpersonal nature of clinical, educational, organizational and community work, and they are associated with key outcomes in the quality of care and the wellbeing of individuals and communities. The literature on social and emotional learning has shown that systematic interventions can promote significant improvements in these skills, while also pointing to persistent challenges in their assessment and in sustaining changes over the long term (Durlak et al., 2011).

Ethical competences involve the ability to recognize responsibilities toward others, anticipate the consequences of interventions, deliberate about dilemmas and act in a contextualized, respectful and reflective manner (Fouad et al., 2009; Kaslow et al., 2009). In community work, professional ethics acquire a particular complexity that goes beyond conventional deontological principles, requiring attention to power relations, institutional conditions, social inequalities and the long-term effects of interventions (Mitchell, 2008). In the Latin American context, community psychology has developed a strong body of reflection on the need for ethically situated practices that are sensitive to the historical conditions of territories and oriented toward social justice (Montero, 2004; Olivares et al., 2012; Pavez et al., 2021; Wiesenfeld, 2014; Winkler et al., 2014). Systematizations of training processes in Community Social Psychology programs at Latin American universities have underlined the need to sustain an ethical-political and affective stance in the university-community relationship, avoiding the instrumentalization of the discipline in local territories (Rojas Lizano and Araya Carvajal, 2024). In this line, ethical reflection in community contexts is understood as an embodied and situated practice that emerges from genuine relationships with communities, rather than from the application of abstract deontological principles (Fernández, 2018). These perspectives provide a normative horizon for assessing the kinds of ethical sensitization that service-learning promotes in early stages of training.

Within this framework, experiences developed in schools and social organizations can foster more complex and contextualized understandings of the professional role, insofar as they expose students to multiple levels of social and institutional interaction, as proposed by ecological perspectives on community intervention (Bronfenbrenner, 1979; Trickett, 2009). However, several authors have warned that community experiences can also reproduce asymmetrical relationships or assistentialist approaches if they are not accompanied by systematic processes of critical reflection and genuine dialog with communities (Gerstenblatt, 2014; Mitchell, 2008). In this study, we focus on service-learning experiences in schools and social organizations as a specific arena where socioemotional and ethical competences can begin to be developed and critically examined.

### 2.3 Evaluating service-learning through mixed-methods designs

The evaluation of service-learning experiences poses methodological challenges because of the multidimensional nature of the learning processes involved, which encompass academic, personal, ethical and relational dimensions (Eyler and Giles, 1999). These forms of learning are difficult to capture through exclusively quantitative strategies or standardized outcome measures, especially when socioemotional, ethical or dispositional competences are at stake. In this context, mixed methods approaches make it possible to integrate general trends derived from quantitative information with qualitative analyses aimed at understanding students’ meanings and subjective experiences (Creswell and Plano Clark, 2018; Verd and López, 2008). This kind of design has been increasingly used in recent studies on service-learning in higher education to capture both the magnitude of change and the processes through which it occurs.

Post-intervention mixed designs, although limited in their capacity to establish causal relationships, can provide valuable evidence on situated formative experiences when their scope and limitations are explicitly acknowledged. Recent reviews of service-learning in higher education have highlighted the need to move toward contextualized evaluations that account for the complexity of the formative processes involved, as well as for the institutional and community conditions that support them (Camilli Trujillo et al., 2021; Tijsma et al., 2023).

At the same time, the literature has pointed to inherent limitations of self-report measures in the evaluation of personal and socioemotional competences, particularly due to social desirability biases, challenges in cross-context comparisons and difficulties in operationalizing complex constructs (Duckworth and Yeager, 2015). However, when combined with qualitative strategies, situated approaches make it possible to access experiential and reflective dimensions that are difficult to capture through standardized instruments. From this perspective, the analytical interest lies not only in identifying formative outcomes, but also in understanding how students interpret, elaborate and attribute meaning to early experiences of approximation to professional practice. In this scenario, evaluations that integrate different types of evidence and go beyond global satisfaction indicators become especially pertinent. A mixed methods design with explicit integration of quantitative and qualitative data offers a way to capture both perceived outcomes and the formative processes that sustain them.

## 3 Methodology

### 3.1 Design

We employed a post-intervention convergent mixed-methods design with an exploratory-descriptive scope. Quantitative and qualitative data were collected simultaneously through a mixed survey and analyzed in a complementary manner. This design was chosen to examine general trends in students’ perceptions of the service-learning experience and, at the same time, to deepen understanding of the meanings they attributed to their socioemotional and ethical learning in community contexts. Because service-learning was integrated as a mandatory curricular component, the study evaluated a single-group cohort without a non-participating control group. Consequently, student evaluations reflect perceived learning following the experience rather than contrafactual causal effects.

### 3.2 Participants

The sample comprised 55 s-year psychology students from a regional university in southern Chile. Their ages ranged from 19 to 37 years (*M* = 22.09, SD = 4.27), with most participants between 19 and 22 years old. In total, 39 students identified as women, 15 as men, and 1 participant preferred not to disclose their gender.

Most students reported studying only (56.4%), whereas 38.2% combined work and study, and a small proportion held part-time jobs or other work situations (5.4%). Regarding educational background, the majority had completed public secondary education (70.9%), followed by technical education (18.2%), with smaller proportions coming from subsidized private and private schools (5.5% each). More than half reported having no previous experience in community or social organizations (54.5%), while 29.1% indicated low, 12.7% moderate, and 3.6% significant prior involvement. Service-learning projects were mainly implemented in community centers (40.0%), universities (34.5%), and schools or high schools (21.8%), with a small number of students participating in other institutional settings (3.6%; see Table 1).

**Table 1:** Sociodemographic characteristics of participants (*N* = 55).

Characteristic	Category	*n*	%
Gender	Women	39	70.9
	Men	15	27.3
	Prefer not to report	1	1.8
Employment status	Study only	31	56.4
	Work and study	21	38.2
	Part-time work and study	2	3.6
	Other	1	1.8
Secondary education type	Public	39	70.9
	Technical	10	18.2
	Subsidized private	3	5.5
	Private	3	5.5
Previous experience in community settings	None	30	54.5
	Low	16	29.1
	Moderate	7	12.7
	Significant	2	3.6
Service-learning setting	Community center	22	40.0
	University	19	34.5
	School/High school	12	21.8
	Other	2	3.6

Participants’ age ranged from 19 to 37 years (*M* = 22.09, SD = 4.27).

All participants were enrolled in modules within the curriculum’s strand on territorial engagement and community intervention. Of these, 19 were taking the module *Territorial Practice and Psychosocial Development* and 36 the module *Territorial Practice and Participatory Methodologies*. Both modules are taught on a semester basis and constitute the students’ first systematic fieldwork experiences in the degree program. Participation in the study was voluntary; written informed consent was obtained, and confidentiality and anonymity in data handling were ensured in accordance with the institution’s ethical guidelines for research involving human participants.

### 3.3 Service-learning context

The service-learning experiences took place in schools and social organizations located in urban and semi-urban areas of a region in southern Chile, within the framework of collaboration agreements between the university and community stakeholders. These projects were carried out over a full academic semester (16 weeks). In these settings, student teams participated in participatory diagnostic activities and brief interventions aimed at promoting participation and psychosocial well-being among different groups. Field activities were articulated with theoretical preparation sessions and with systematic supervision and reflection spaces in the classroom, where frameworks from community psychology, participatory approaches, and ethical criteria for working with communities were reviewed. In this way, the practical experiences were explicitly integrated into the training process, functioning as a scaffold for the development of socioemotional and ethical learning in the early stages of psychology education.

### 3.4 Instrument

An *ad hoc* mixed survey was designed to evaluate, in a situated manner, the impact of service-learning on relevant formative dimensions, following recommendations for the assessment of non-cognitive competences in higher education (Duckworth and Yeager, 2015). The development process unfolded in four phases. First, the evaluation dimensions were defined by articulating the course learning outcomes, the principles of service-learning, and conceptual frameworks on socioemotional competences and professional ethics. This process led to 10 dimensions: conceptual learning, participatory diagnosis, psychosocial intervention, socioemotional and collaborative competences, critical reflection and analytical thinking, ethical and professional responsibility, community impact, personal change and professional motivation, service-learning methodology, and context and feasibility.

Second, items were written following criteria of behavioral formulation, contextualization in territorial practice experiences, and the use of language accessible to students in the early stages of training, in order to enhance the ecological validity of the instrument. Third, the items were reviewed by the course teaching team, and adjustments were made according to criteria of clarity, relevance, and coherence with the formative objectives. The final instrument comprised 57 items rated on a 5-point Likert scale (1 = strongly disagree; 5 = strongly agree) and four open-ended questions, integrating quantitative and qualitative components in line with contemporary approaches to formative assessment in higher education (Boud and Falchikov, 2007). For analysis, mean scores were calculated for each dimension based on their corresponding items. To mitigate potential social desirability bias associated with self-report measures in professional ethics and socioemotional skills, survey completion was voluntary, strictly anonymous, and unlinked to course evaluation.

Internal consistency was estimated using Cronbach’s alpha coefficient for each dimension and for the global score. The resulting coefficients indicated acceptable to excellent reliability overall, supporting the internal consistency of the instrument in this context (see Table 2). In addition, one negatively worded item within the context and feasibility dimension was reverse-coded so that higher scores consistently reflected more favorable contextual conditions.

**Table 2:** Students’ perceptions of the service-learning experience: means, standard deviations, 95% confidence intervals and internal consistency by dimension.

Dimension	*k*	*M*	SD	95% CI for *M*	*α*
Conceptual learning	7	4.16	0.57	4.01–4.31	0.94
Participatory diagnosis	5	4.00	0.64	3.82–4.18	0.81
Psychosocial intervention	5	3.79	0.69	3.61–3.97	0.80
Socioemotional and collaborative competences	7	4.05	0.56	3.90–4.20	0.94
Critical reflection and analytical thinking	4	4.25	0.62	4.08–4.42	0.80
Ethical and professional responsibility	6	4.50	0.60	4.34–4.66	0.95
Community impact	6	4.31	0.78	4.09–4.53	0.93
Personal change and professional motivation	8	4.05	0.73	3.86–4.24	0.86
Service-learning methodology	6	4.29	0.67	4.11–4.47	0.80
Context and feasibility	3	4.50	0.61	4.34–4.66	0.75
Global score	57	4.17	0.53	4.03–4.31	0.97

Scores were obtained on a 5-point Likert scale ranging from 1 (strongly disagree) to 5 (strongly agree). *k*, number of items; *M*, mean; SD, standard deviation; 95% CI, 95% confidence interval for the mean; *α*, Cronbach’s alpha.

To examine the internal structure of the instrument, an exploratory factor analysis was conducted on the 57 items using minimum residual extraction with oblimin rotation. The results supported a strong general factor together with a set of correlated dimensions broadly consistent with the 10 theoretical dimensions proposed. Given the relatively small sample size in relation to the number of items, these factor-analytic findings were interpreted cautiously as preliminary evidence of construct validity.

### 3.5 Data analysis

**Figure 1:**
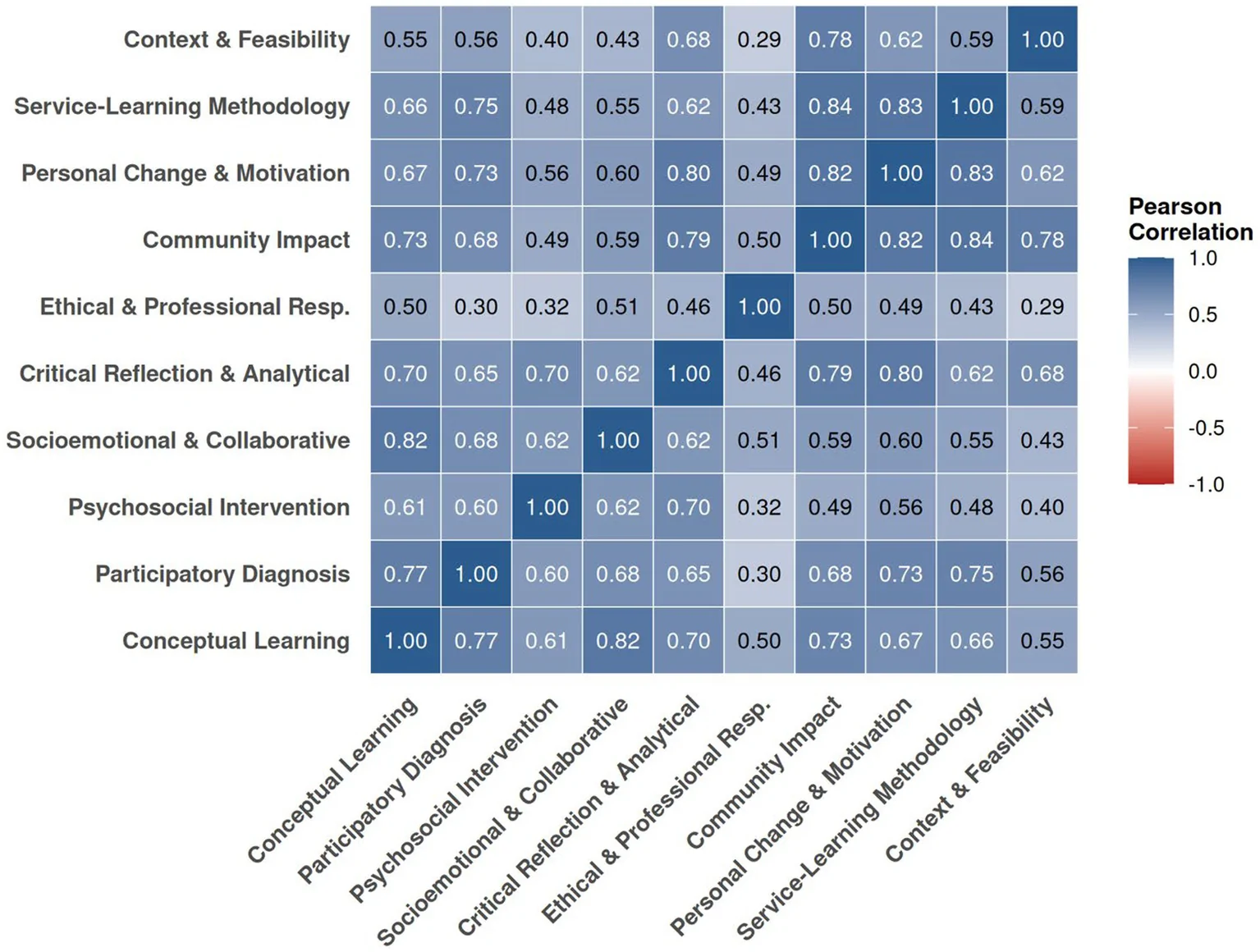
Heatmap of Pearson correlations between the 10 learning dimensions in the service-learning course.

Quantitative data were processed and analyzed using R version 4.3.3 in a Linux environment. The analysis proceeded in three stages. First, descriptive statistics were calculated for each of the 10 learning dimensions and the global score, including means, standard deviations, and internal consistency coefficients (Cronbach’s alpha). These results are presented in Table 2, and the profile of mean scores across dimensions is illustrated in Figure 1.

Second, Pearson correlations among the dimensional indices were examined to explore the pattern of associations between formative dimensions; these results are displayed in Figure 1. Independent-samples *t*-tests with Cohen’s *d* effect sizes and 95% confidence intervals were also conducted to compare scores across selected sociodemographic groupings, with particular attention to previous community experience.

Third, multiple linear regression models were estimated to examine whether direct learning dimensions predicted selected civic, socioemotional, and vocational outcomes. Qualitative data from the four open-ended questions were analyzed through inductive thematic analysis following Braun and Clarke (2006): familiarization with the data, generation of initial codes, construction of themes, review of themes, definition and naming of themes, and production of the report.

To strengthen qualitative rigor, both authors, who were members of the teaching team, independently coded a subsample of responses and then compared their coding, discussing and resolving discrepancies by consensus. Credibility and confirmability were further supported through triangulation between quantitative and qualitative data sources (Creswell and Plano Clark, 2018; Noreña et al., 2012). In the final integration phase, quantitative and qualitative findings were compared and jointly interpreted to identify convergences, complementarities, and tensions between general patterns of perceived learning and students’ articulated meanings.

## 4 Results

This section first presents quantitative findings for the 10 learning dimensions, then qualitative themes derived from the open-ended questions, and finally an integrated interpretation of both strands of data.

### 4.1 Quantitative results

Table 2 summarizes students’ perceptions of the service-learning experience across the 10 learning dimensions, including number of items, means, standard deviations and internal consistency coefficients. Mean scores were consistently in the medium-to-high range (around 4 on a 1–5 scale), with relatively low dispersion, indicating an overall positive and homogeneous evaluation of the course. Ethical and professional responsibility and context and feasibility showed the highest mean scores, followed by community impact and critical reflection and analytical thinking, whereas conceptual learning and psychosocial intervention obtained comparatively more moderate means. This descriptive profile, also illustrated in Figure 1, suggests that students perceived the experience as particularly strong in ethical, reflective and contextual domains, while its contribution to more technical or intervention-related competences, although positive, was somewhat less pronounced.

Beyond mean levels, inter-correlations between the 10 dimensions were uniformly positive and mostly high, indicating that the instrument captures a strongly integrated set of formative outcomes rather than isolated components. In practical terms, students who reported higher development in one area (for example, ethical and professional responsibility) tended to report similarly high development in others, such as socioemotional and collaborative competences, critical reflection or perceived community impact. This pattern is visually represented in Figure 1, where the heatmap of Pearson correlations shows a dense network of moderate to strong associations across dimensions, reinforcing the interpretation of the service-learning course as a holistic formative process.

To explore how these perceived effects were distributed across different student profiles, subscale scores were examined according to selected sociodemographic variables. Comparisons by gender, employment status and secondary school type did not reveal statistically significant differences in any of the 10 dimensions or in the global score (*t*s < 1.15, *p*s > 0.26, *d*s < 0.32), indicating that the course was evaluated in a broadly similar way across these groups. By contrast, previous participation in community or social organizations emerged as a more influential factor: students with some prior community experience showed significantly higher scores in conceptual learning than those without such experience, with medium effect sizes (*t*(53) = 2.47, *p* = 0.018, Cohen’s *d* = 0.67, 95% CI [0.12, 1.22]), and similar tendencies were observed for participatory diagnosis (*t*(53) = 1.99, *p* = 0.053, Cohen’s *d* = 0.54, 95% CI [−0.00, 1.08]) and personal change and motivation (*t*(53) = 1.88, *p* = 0.064, Cohen’s *d* = 0.51, 95% CI [−0.03, 1.05]). These patterns suggest that previous community engagement may facilitate the assimilation of academic content and the perceived impact of the projects, although the modest sample size calls for cautious interpretation of these effects.

Taken together, the quantitative findings indicate that the service-learning experience was perceived as especially powerful for promoting ethical responsibility, socioemotional competences, critical reflection and community engagement, while its contribution to conceptual learning and technical psychosocial intervention competences, although clearly positive, was comparatively more moderate. In early stages of training, this differential profile is consistent with the idea that service-learning functions primarily as a space for sensitization, reflection and initial engagement with communities, rather than as a context for demonstrating fully consolidated intervention skills.

### 4.2 Qualitative results

The thematic analysis of the open-ended responses allowed us to identify five themes that deepen and nuance the trends observed in the quantitative data. As shown in Table 3, each theme is presented with a brief description, its related quantitative dimension(s) and a representative student quote.

**Table 3:** Themes emerging from the thematic analysis of students’ service-learning experience.

Theme	Description	Related quantitative dimension(s)	Illustrative quote
Theory-practice articulation	Connection between community/educational psychology content and the design of contextually relevant interventions.	Conceptual learning; Psychosocial intervention	“Getting to know the experience of working with a group helped me create interventions that were relevant to the context where we worked.”
Teamwork and socioemotional skills	Development of skills for collaboration, communication, decision-making and conflict resolution within the team.	Socioemotional and collaborative competences	“The most important learning was working in a team; it made communication and decision-making easier.”
Ethical sensitization and empathy	Increased awareness of the impact of interventions, the importance of active listening, and respect for people’s boundaries.	Ethical and professional responsibility; Community impact	“Connecting with the students and practicing active listening made them feel genuinely heard.”
Contextual understanding of problems	Recognition of the diversity of problems, personalities and contexts, and the need to adapt interventions to the territory.	Context and feasibility; Community impact	“I saw different problems and personalities; this helped me personalize everything and pay more attention to community contexts.”
Approximation to the professional role and limits	Rehearsal of the psychologist role, combined with awareness of insecurities and the need for more tools and organization.	Socioemotional and collaborative competences; Conceptual learning; Psychosocial intervention	“Socioemotional skills are what I take away the most, but I want to improve; I would like to intervene with more people and be less individualistic.”

Quotes are translated from Spanish to United Kingdom English to protect students’ anonymity and facilitate readability.

The first theme, theory-practice articulation, refers to the possibility of “seeing in reality” the content addressed in the courses, especially that related to community, psychosocial and educational psychology. This articulation was expressed both in the understanding of institutional and territorial dynamics and in the identification of psychosocial factors that affect the everyday lives of communities. The experience was described as an opportunity to “put theoretical frameworks to the test” and to recognize their scope and limits in concrete contexts, reinforcing but also qualifying the medium-to-high scores obtained in the conceptual learning dimension.

The second theme, teamwork and socioemotional skills, captures the development of collaborative, communicative and conflict-resolution skills within the student teams. Students highlighted teamwork as a key space for developing communication, coordination and collective decision-making, which they considered fundamental for their future professional practice. These narratives resonate with the high mean in the socioemotional and collaborative competences subscale and help illustrate how these competences are enacted in practice, for example through the management of disagreements, the distribution of tasks or the construction of supportive relational climates within the teams.

The third theme, ethical sensitization and empathy, reflects increased awareness of the impact of interventions, the importance of active listening and the need for care when working with children, adolescents and adults. Students mentioned concerns about the possible effects of their interventions, the position of the university in local territories and power differentials between the institution and communities, which contributed to greater awareness of the ethical implications of psychosocial work. This theme deepens the very high scores observed in ethical and professional responsibility, showing that such evaluations are not only abstract but grounded in concrete dilemmas, reflections and emotional responses.

The fourth theme, contextual understanding of psychosocial problems, reflects recognition of the complexity of the institutional and territorial conditions in which communities live, as well as of the multiple familial, school, community and structural dimensions that influence the situations addressed, in line with ecological perspectives on community intervention (Bronfenbrenner, 1979; Trickett, 2009). This understanding was associated with a questioning of individualizing views of problems and with greater recognition of contextual and structural factors, which complements the high scores in community impact and context and feasibility by showing how students make sense of the territories in which they intervene.

The fifth theme, initial approximation to the professional role and formative limits, describes the experience as an opportunity to rehearse future psychologist roles in real settings, which generated both motivation and a sense of purpose, as well as feelings of technical insecurity. Students reported needing more conceptual and methodological preparation to plan interventions, manage emerging situations and make decisions in contexts of uncertainty, which resonates with the more moderate scores in conceptual learning and psychosocial intervention. These accounts suggest that, while service-learning opens a valuable space for role experimentation, it also exposes perceived gaps in technical preparation.

### 4.3 Integration of results

The integration of quantitative and qualitative findings (Tables 2 and 3) indicates that the service-learning experience was perceived as a particularly powerful formative device for developing ethical sensitization, socioemotional competences, critical reflection and community engagement among psychology students in the early stages of their training. High scores in ethical and professional responsibility, community impact, the service-learning methodology and socioemotional and collaborative competences are reinforced by narratives that highlight increased awareness of the university’s role in local territories, the questioning of assistentialist positions, and the centrality of teamwork and affective climates in community work. At the same time, the high mean in the context and feasibility dimension is largely consistent with students’ descriptions of supportive relationships with schools, community centers and social organizations, alongside recognition of institutional constraints and territorial inequalities.

The very high inter-correlations among the 10 quantitative dimensions further support an interpretation of the service-learning course as a holistic formative experience, in which cognitive, socioemotional, ethical and civic components tend to develop in tandem rather than in isolation. In this sense, the instrument appears to capture not only specific competences but also a broader configuration of dispositions that are activated as students participate in community-based projects, engage with institutional actors and negotiate their own position as future professionals.

By contrast, the more moderate scores in conceptual learning and psychosocial intervention, together with narratives about technical insecurity and the need for greater preparation, suggest that the service-learning experience contributed more decisively to the development of ethical, socioemotional and reflective dispositions than to the consolidation of disciplinary knowledge or technical intervention competences. Students’ accounts of struggling to translate theoretical frameworks into action, managing unexpected situations and making decisions in contexts of uncertainty nuance the generally positive quantitative evaluations and point to a differential pattern of formative contributions. This profile underscores the importance of understanding service-learning in early training as a space of sensitization, approximation to professional roles and construction of ethical and relational dispositions, rather than as an instance for demonstrating fully consolidated technical competences.

## 5 Discussion

The results of this study indicate that the service-learning experience was perceived as particularly powerful for the development of ethical responsibility, socioemotional and collaborative competences, critical reflection and community engagement, whereas its contribution to conceptual learning and technical intervention competences was evaluated in more moderate terms. This pattern addresses RQ1 and is consistent with meta-analyses showing that service-learning tends to generate more robust effects in socioemotional and civic engagement dimensions than in strictly cognitive indicators (Celio et al., 2011; Conway et al., 2009; Yorio and Ye, 2012). In the field of psychology education, these findings reinforce the idea that early experiences in community settings function primarily as devices for sensitization and reflection on the professional role rather than as instances for consolidating advanced competences (Bringle et al., 2016; Valdez and Lovell, 2022). The strong and generalized positive evaluations observed across the 10 dimensions, together with their high inter-correlations, are coherent with this view of service-learning as a holistic formative device that tends to activate multiple dispositional and relational components at once, rather than narrowly delimited skills. Recent service-learning studies in higher education report similar patterns; for instance, Sánchez-Jiménez et al. (2025) found that a mixed-methods service-learning program for pre-service teachers was associated with significant gains in specific components of resilience, and Xiao (2026) showed that higher perceived quality of service-learning experiences was positively related to students’ sense of community, prosocial behavior and social responsibility, with mean scores in a similar medium-to-high range.

Addressing RQ2, the high scores in ethical responsibility and community impact, together with qualitative accounts emphasizing concerns about “not doing harm,” awareness of community expectations and questioning of assistentialist positions, speak directly to the Latin American community psychology tradition, which has insisted on the centrality of situated ethics, power relations and social justice (Montero, 2004; Olivares et al., 2012; Pavez et al., 2021; Wiesenfeld, 2014; Winkler et al., 2014). Convergently, systematizations of training processes in Community Social Psychology at other Latin American universities highlight the need to sustain an ethical-political stance and an affective praxis in the university-community relationship (Rojas Lizano and Araya Carvajal, 2024). From this perspective, the experience analyzed here seems to foster processes of awareness regarding the university’s position in local territories and the ethical implications of psychosocial work in communities, which is especially relevant in regional universities where proximity to vulnerable contexts intensifies these dimensions of active learning. The convergence between elevated scores and students’ narratives suggests that the course contributes less to isolated “ethical competences” and more to a broader ethical-political disposition toward community work, in line with these regional traditions.

The importance that students attributed to the articulation between theory and practice and to a contextual understanding of psychosocial problems is consistent with competence-based training models that conceive professional development as a process of progressive integration of knowledge, skills, attitudes and reflective capacities (Fouad et al., 2009; Kaslow et al., 2009; Rodolfa et al., 2005). In the early stages of training, it is to be expected that the emphasis falls on the development of socioemotional and ethical dispositions, such as empathy, perspective taking and sensitivity to diversity, rather than on the mastery of complex technical interventions, in line with international guidelines on developmental trajectories of competences in psychology (American Psychological Association, 2023). The fact that these emphases appear both in quantitative scores and in students’ narratives suggests that the course effectively supports the progressive integration of knowledge, skills, attitudes and reflective capacities envisaged in competence-based models, especially in relation to relational and contextual dimensions of professional development.

The relatively lower scores in conceptual learning and psychosocial intervention, together with narratives referring to feelings of technical insecurity and the need for greater preparation, highlight a well-known tension in the service-learning literature: the gap that may exist between intense, meaningful experiences for students and the explicit integration of these experiences with core curricular content and skills (Eyler and Giles, 1999; Sturgill and Motley, 2014). These results suggest that, while service-learning offers a privileged context for the development of ethical sensitization and critical reflection, its contribution to the consolidation of disciplinary knowledge and technical competences depends on a pedagogical design that ensures systematic articulation between field-based activities, conceptual seminars and structured supervision. The descriptive analyses by student profiles also point in this direction: although the experience is generally valued across different sociodemographic groups, there are indications that prior community involvement may be associated with slightly higher perceived gains in some dimensions, which underscores the need to scaffold more explicitly the translation between lived experience and conceptual frameworks for those with less initial familiarity with community work. Recent studies evaluating teaching-service models in psychology report similar challenges, identifying discrepancies between high overall valuation of the experiences and more modest perceptions of mastery of specific clinical competences (Roncancio-Moreno et al., 2025).

From a methodological standpoint, the combination of quantitative and qualitative data made it possible to capture both general patterns in students’ perceptions and nuances and tensions that would have been unlikely to emerge through exclusively standardized strategies. This approach directly addresses RQ3 and is consistent with recommendations that advocate the use of mixed methods and situated evaluations to account for the complexity of formative processes associated with service-learning in higher education (Camilli Trujillo et al., 2021; Creswell and Plano Clark, 2018; Tijsma et al., 2020). At the same time, the findings confirm the limitations of self-report measures for evaluating socioemotional and ethical competences: triangulation with qualitative data nuanced the reading of the high scores in ethical responsibility and socioemotional competences, showing that they coexist with an acute awareness of students’ own formative limits. Without this triangulation, a purely quantitative reading might have overestimated the level of competence achieved. Moreover, the very high inter-correlations between subscales invite caution when interpreting each dimension as fully distinct at the empirical level and highlight the added value of qualitative data to differentiate how students actually experience and enact these intertwined competences in specific community contexts.

In terms of implications for the design of service-learning as an active methodology, the results support its potential to introduce students early into community work experiences that foster the construction of ethical and socioemotional sensitivity, aligned with contemporary challenges of the profession. However, they also point to the need to articulate these experiences with devices that strengthen conceptual learning and technical intervention competences, such as theory-practice integration seminars, structured supervision and formative assessment coherent with professional competence frameworks (Durlak et al., 2011; Fouad et al., 2009; Kaslow et al., 2009). Designing these devices in ways that explicitly address the tensions identified here, for example by incorporating guided reflection on moments of technical insecurity, joint analysis of cases that link fieldwork situations with theoretical models, and feedback spaces where students can rehearse decision-making in complex scenarios, could help transform service-learning from a primarily sensitizing experience into a more robust platform for progressive competence consolidation. In the context of Latin American regional universities, such devices can contribute both to the quality of training and to the consolidation of reciprocal, sustainable links with communities (Montero, 2004; Sandoval et al., 2025; Wiesenfeld, 2014).

Several limitations of this study must be acknowledged. The relatively small sample, restricted to a single cohort from a regional university, limits the generalizability of the findings and calls for caution when extrapolating them to other institutional contexts. Although this sample size limits the statistical generalizability of the findings, it represents the entire active cohort of students enrolled in the program during the study period, capturing the total available population for this curricular innovation. The post-intervention design without a comparison group prevents causal inferences and places the study in line with many exploratory investigations on service-learning in higher education (Narong and Hallinger, 2023; Tijsma et al., 2020). In addition, the use of self-report measures increases the likelihood of social desirability bias, particularly in ethical and socioemotional dimensions (Duckworth and Yeager, 2015). Some subscales, especially the brief context and feasibility subscale and the refined socioemotional subscale, also showed only modest internal consistency, so their scores should be interpreted with caution. The strong convergence between subscales, while theoretically interpretable as a marker of holistic development, also suggests potential redundancy between some dimensions at the measurement level and points to the need for future refinement of the instrument, for instance by revising items that may be capturing very similar aspects of students’ experiences. Taken together, these limitations open a fertile field for future research that uses longitudinal designs, incorporates the perspectives of community partners and instructors, and combines self-report with performance-based and observational assessments. Such studies could also examine how different service-learning designs, varying in duration, intensity of community contact and degree of integration with other curricular components, shape the balance between ethical and socioemotional development and technical competence acquisition over time.

At the same time, the instrument used and its combination with thematic analysis configure an evaluation model that can be reused and adapted in other training contexts, especially where there is interest in differentiating between conceptual learning, socioemotional competences, ethical responsibility and community impact. Beyond the global satisfaction measures frequently used in service-learning research, this multidimensional tool distinguishes between conceptual, socioemotional, ethical, community impact and contextual dimensions, which are rarely assessed jointly in evaluation instruments. By combining Likert-type subscales with open-ended questions analyzed through thematic analysis, it offers a simple yet robust mixed-methods template that other psychology programs can tailor to their own territorial and institutional realities. In this sense, the present study contributes not only substantive evidence about the formative potential and limits of service-learning in a regional psychology program, but also a concrete, adaptable framework for systematically evaluating similar experiences in other higher education settings.

## 6 Conclusion

The service-learning experience analyzed in this study was perceived by second-year psychology students as particularly meaningful for the development of ethical sensitization, socioemotional and collaborative competences, critical reflection and community engagement, in line with evidence highlighting the impact of service-learning on attitudinal and socioemotional dimensions in higher education. Given the non-experimental design, these findings should be interpreted as perceived learning outcomes rather than direct causal effects. The consistently high scores observed in these dimensions, together with their strong interrelations, point to a pattern of holistic development rather than to isolated gains in specific skills, suggesting that in early stages of training service-learning functions primarily as a device for initial approximation to the professional role and for the construction of ethical and relational dispositions, rather than as a space for consolidating advanced technical competences.

The more moderate scores in conceptual learning and psychosocial intervention, together with narratives referring to feelings of technical insufficiency, indicate that participation in community experiences alone is not enough to strengthen disciplinary knowledge and intervention skills. Consequently, it becomes necessary to articulate service-learning with theory–practice integration seminars, specialized supervision spaces and formative assessment devices that are coherent with professional competence frameworks in psychology. Designing these complementary components so that they explicitly address the tensions and uncertainties experienced by students, through guided reflection on difficult situations, structured case discussions and feedback on decision making in complex contexts, may help transform service-learning from a primarily sensitizing experience into a more robust platform for progressive competence consolidation.

From a methodological perspective, the convergent mixed-methods design made it possible to combine an overarching view of students’ perceptions with a deeper understanding of the meanings they attributed to the experience, in line with recommendations that advocate mixed-methods approaches to evaluate complex formative processes in higher education. At the same time, the small sample size, the contextual nature of the experience, the use of self-report measures and the modest internal consistency of some subscales, particularly the brief context and feasibility subscale and the refined socioemotional subscale, limit the generalizability of the findings and suggest that these dimensions should be interpreted with caution. The strong convergence between subscales also invites prudence when interpreting each dimension as empirically distinct and points to the need for further psychometric refinement of the instrument, for example by revising or expanding items that may be tapping very similar facets of students’ experiences. These limitations underscore the need for longitudinal, comparative and multimethod studies that examine in greater detail the development of socioemotional and ethical competences in psychology training, as well as the specific contribution of different service-learning designs to the balance between ethical and socioemotional development and technical competence acquisition over time.

In the context of Latin American regional universities, this study provides situated evidence on the potentials and challenges of service-learning in early psychology education, showing both its contribution to the construction of ethical and community-oriented sensitivity and the importance of strengthening its curricular integration and the psychometric refinement of evaluative tools. By offering an instrument that differentiates between conceptual, socioemotional, ethical, community impact and contextual dimensions and combining it with thematic analysis of open-ended responses, the study also proposes a simple, adaptable framework for evaluating similar experiences in other programs and institutions. These results may inform decisions regarding course design, planning of field-based placements and the development of formative assessment strategies that more closely align academic training with the needs and dynamics of the territories in which the university is embedded.

## Data Availability

The raw data supporting the conclusions of this article will be made available by the authors, without undue reservation.
